# Ruptured Ectopic Pregnancy in the Presence of an Intrauterine Device

**DOI:** 10.5811/cpcem.2019.1.41345

**Published:** 2019-01-22

**Authors:** Matthew R. Neth, Maxwell A. Thompson, Courtney Blayke Gibson, John P. Gullett, David C. Pigott

**Affiliations:** University of Alabama at Birmingham, Department of Emergency Medicine, Birmingham, Alabama

## Abstract

Ruptured ectopic pregnancy is the leading cause of first trimester maternal mortality. The diagnosis of ectopic pregnancy should always be suspected in patients with abdominal pain, vaginal bleeding or syncope. While the use of an intrauterine device (IUD) markedly reduces the incidence of intrauterine pregnancy, it does not confer equal protection from the risk of ectopic pregnancy. In this report we discuss the case of a female patient who presented with a ruptured ectopic pregnancy and hemoperitoneum despite a correctly positioned IUD.

## INTRODUCTION

Although many forms of contraception are available worldwide, including oral hormonal contraceptives, barrier devices and others, the intrauterine device (IUD) remains a popular choice. By 2015 estimates, IUD use reached greater than 100 million women worldwide, and IUDs are used by approximately 7% of reproductive-age women in the United States (U.S.).^1^,^2^ The two major types currently available here are the T-shaped copper IUD introduced in the late 1980s and the similarly-shaped levonorgestrel-releasing IUD introduced in the early 2000s.^2^ While it is clear that IUD use reduces the overall rate of pregnancy (including ectopic gestation), for patients with IUD failure (i.e., unintended pregnancy), the presence of an IUD markedly increases the risk that such a pregnancy will be extrauterine. A recent multicenter study from China demonstrated that pregnancies during IUD use were highly likely to be ectopic (adjusted odds ratio +16.4) and accounted for ~10% of ectopic pregnancies in the studied population.^3^

Although the overall incidence of ectopic pregnancy is approximately 2%,^4^ the incidence of ectopic pregnancy in emergency department (ED) patients has been shown to be significantly higher. Among patients presenting to the ED with either abdominal pain or vaginal bleeding, or both complaints, ectopic pregnancy rates has been reported as high as 6–16%.^5^ Ectopic pregnancy continues to be associated with significant maternal risk, with mortality estimates ranging from 3–6%, with the majority of deaths resulting from hemorrhage.^6^,^7^

We report a case of ruptured ectopic pregnancy in a patient with an IUD. Rapid use of point-of-care ultrasonography (POCUS) enabled a timely diagnosis and potentially life-saving treatment of a patient in whom the diagnosis of pregnancy was thought to be extremely unlikely.

## CASE REPORT

A 34-year-old woman with no significant past medical history presented to our ED with acute onset of suprapubic pain two hours prior to arrival. Pain was sharp, constant and non-radiating with associated nausea and vomiting. She denied any fever, vaginal bleeding, vaginal discharge, dark or bloody stools, flank pain, dyspnea, or syncope. The patient reported no history of pelvic inflammatory disease (PID) or ectopic pregnancy. She stated that she had a copper IUD placed approximately three years prior. Her initial vital signs included a blood pressure of 140/81 millimeters of mercury, pulse of 96 beats per minute, respiratory rate of 20 breaths per minute, and temperature of 98.1° Fahrenheit. Physical examination was remarkable for moderate to severe lower abdominal tenderness to palpation with associated rebound and guarding.

Although a urine pregnancy test was ordered shortly after the patient arrived, while walking to the restroom, the patient sustained an episode of lightheadedness and near-syncope. Immediately following this episode, point-of-care transabdominal pelvic sonography was performed to further evaluate the etiology for the patient’s presentation.

**Image 1:**
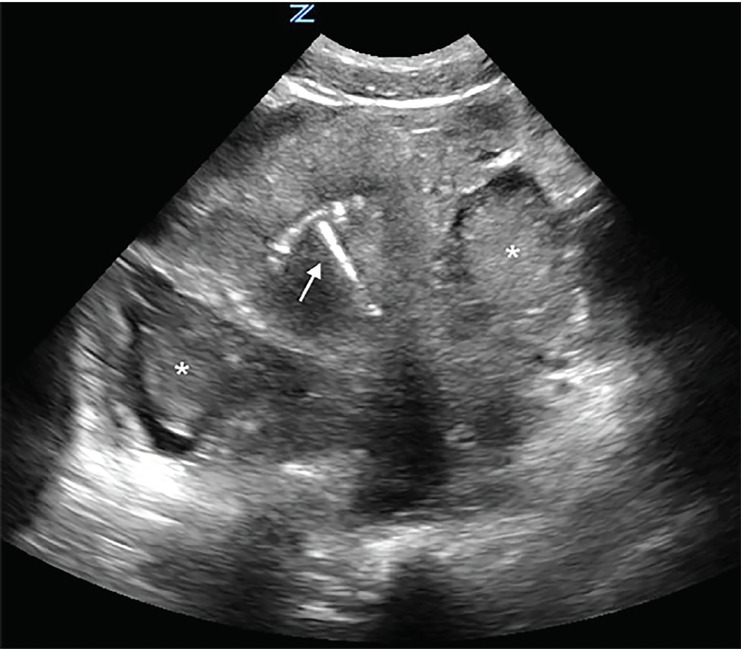
Transabdominal transverse ultrasound view of the pelvis. Note presence of intrauterine device within uterus (arrow) and pelvic hematoma (asterisks).

**Image 2:**
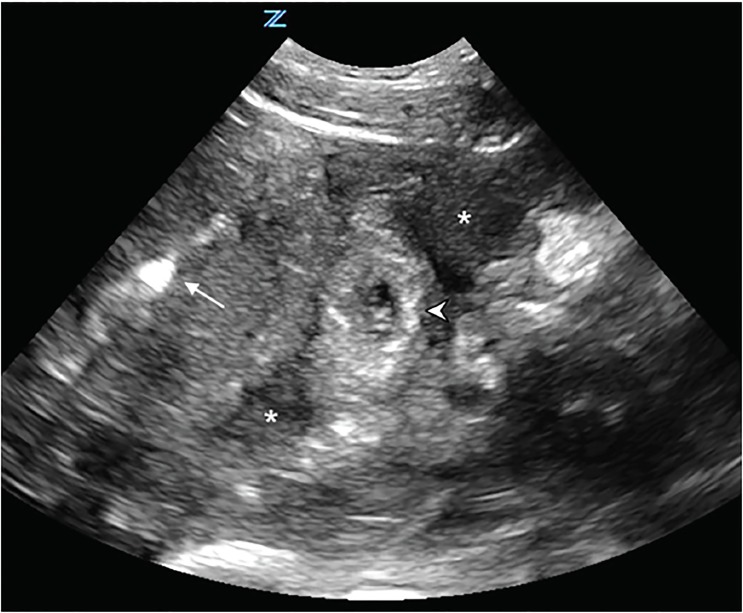
Transabdominal transverse ultrasound view of the pelvis. Note presence of intrauterine device within uterus (arrow) and adjacent ectopic pregnancy in left adnexa (arrowhead). Hypoechoic fluid (asterisks) surrounds the uterus and adnexa.

**Image 3:**
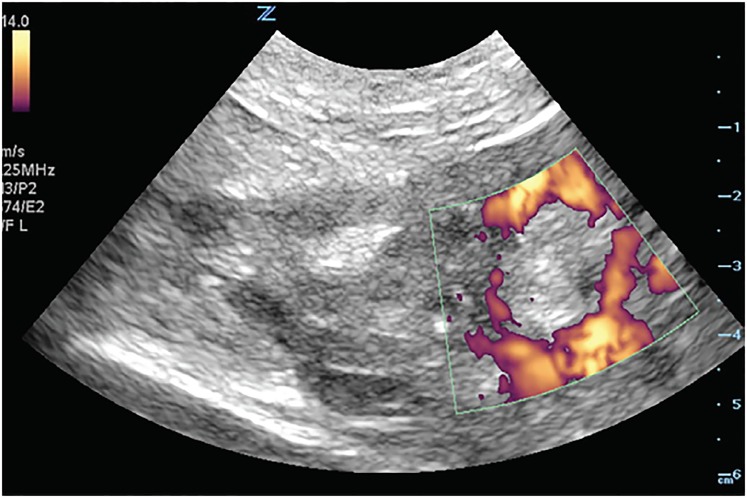
Transabdominal power Doppler ultrasound of the left adnexa. Note ectopic pregnancy with “ring of fire” sign reflecting peripheral hypervascularity.

A focused assessment with sonography in trauma (FAST) protocol revealed free fluid in Morison’s pouch and the splenorenal space, as well as in the pelvis. Transabdominal pelvic sonography also showed evidence of an IUD within the uterus without evidence of an intrauterine pregnancy. Extensive pelvic hematoma was noted surrounding the uterus (Image 1, Video 1). Transabdominal ultrasound examination of the adnexa showed a thick-walled circular structure in the left adnexa (Image 2) demonstrating marked hypervascularity (“ring of fire” sign) (Image 3,Video 2) as well as fetal cardiac activity consistent with a live ectopic pregnancy. Given these findings, emergent gynecology consultation was obtained. Initial laboratory studies showed mild anemia and leukocytosis (hemoglobin 10.9 grams per deciliter, white blood cell count 12.4 × 10^9^ per liter). Serum beta-human chorionic gonadotropin was 24,976 milli-international units per milliliter. The patient was taken emergently to the operating room where a ruptured left tubal ectopic pregnancy with one liter hemoperitoneum was noted, and salpingectomy was performed. The patient remained hemodynamically stable, and was subsequently discharged in good condition.

**CPC-EM Capsule**

What do we already know about this clinical entity?

*Factors affecting fallopian tube or uterine function, such as prior surgery, infection, or instrumentation, may increase risk for ectopic pregnancy*.

What makes this presentation of disease reportable?

*This case illustrates the utility of point-of-care ultrasound in the diagnosis of ectopic pregnancy in a setting where pregnancy was thought to be very unlikely*.

What is the major learning point?

*In this case, a ruptured ectopic pregnancy was diagnosed in a patient with an intrauterine device, an important risk factor for ectopic pregnancy*.

How might this improve emergency medicine practice?

*This case reinforces the importance of a high clinical suspicion for ectopic pregnancy in reproductive-age women, despite the use of highly effective contraception*.

## DISCUSSION

POCUS has long been shown to be a valuable tool for the emergency physician, particularly in the evaluation of patients with early pregnancy. This cse demonstrates the utility of POCUS in the rapid, accurate diagnosis of ruptured ectopic pregnancy leading to definitive treatment in a patient on highly effective contraceptive therapy. The presence of ruptured ectopic pregnancy with concurrent IUD use is notable, as this complication has rarely been reported.

One of the most attractive features of the IUD is its proven efficacy in preventing pregnancy. The one-year failure rate for the copper and the levonorgestrel-releasing IUD has been reported at 0.8 and 0.1 unintended pregnancies per 100 women, respectively.^8^ Although IUD use markedly reduces the overall rate of pregnancy (including ectopic pregnancy) compared to patients not on contraception, in patients with IUD failure (i.e., unintended pregnancy during IUD use), the risk of ectopic pregnancy ranges from 15–27%.^9^ As previously noted, the incidence of ectopic pregnancy in the U.S. is approximately 2%.^4^

While it is clear that IUD use can reduce the overall rate of pregnancy, for patients with IUD failure the presence of an IUD markedly increases the risk that such a pregnancy will be extrauterine. Intrauterine pregnancy in the setting of reported IUD use is rare, and is three times more likely in patients with a malpositioned or inadvertently missing IUD.^10^ While IUDs are clearly effective in the prevention of intrauterine pregnancy, they are not necessarily designed to prevent extrauterine gestation. Although the overall incidence of ectopic pregnancy in IUD patients is very low, it is clear that those who become pregnant in the setting of IUD use are at increased risk for ectopic pregnancy.^3^,^8^,^11^,^12^

Ectopic pregnancy is defined as the implantation of a fertilized ovum outside the endometrial cavity.^13^ Multiple risk factors for ectopic pregnancy have been identified, including age, history of PID, smoking, previous ectopic pregnancy, and in vitro fertilization.^14^,^15^ POCUS is a valuable tool in the evaluation of the patient with suspected ectopic pregnancy. While transabdominal ultrasound can rapidly demonstrate the presence of significant intraperitoneal hemorrhage, transvaginal ultrasound (TVUS) is considered the imaging modality of choice for the definitive diagnosis of ectopic pregnancy, allowing for earlier visualization and diagnosis.^16^ The sensitivity of TVUS for ectopic pregnancy has been reported at greater than 90%.^17^ The accuracy and utility of TVUS, however, may vary depending on operator experience, maternal body mass index, fibroids, and ovarian pathology.^16^ In patients where a clear ectopic pregnancy can be visualized, as in our case, transabdominal ultrasound may also provide a definitive diagnosis.

Apart from direct visualization of an ectopic pregnancy, the POCUS evaluation of the patient with suspected ectopic pregnancy may also yield additional evidence suggestive of ectopic pregnancy. Other helpful signs include an empty uterus, adnexal mass, free fluid, or the pseudogestational sac of ectopic pregnancy. While useful, these signs alone do not have reported sensitivities high enough to effectively rule out ectopic pregnancy based on current literature.^18^ The most concerning ultrasound finding in this setting, the presence of free intraperitoneal fluid in Morison’s pouch, has been found to be predictive of the need for operative intervention.^19^ With this finding, the emergency physician can use ultrasound as a means to expedite patient care and reduce the risk for hemodynamic compromise due to ongoing intraperitoneal hemorrhage.

Pregnant patients with an empty uterus on ultrasound, but without clear signs of ectopic pregnancy such as an extrauterine gestational sac, adnexal mass or free fluid, are classified as having a pregnancy of unknown location. These patients will require follow-up until their pregnancy location is confirmed. Ultimately, approximately 7–20% of women with an initial pregnancy of unknown location will eventually receive a diagnosis of an ectopic pregnancy.^13^,^16^

This case report is unique in that it demonstrates the rapid identification of an ectopic pregnancy in a patient with a concurrent IUD using POCUS by EPs. Although the patient’s history of current IUD use initially suggested that pregnancy was unlikely, this case clearly demonstrates that a standard ultrasound-based approach to the ED evaluation of the patient with early pregnancy provided a rapid definitive diagnosis of an emergent medical condition.

## CONCLUSION

Although the use of POCUS in the evaluation of a woman presenting with acute pelvic pain has been well-described, the complicating factor of IUD use in the setting of early pregnancy makes this case notable. Point-of-care transabdominal pelvic ultrasound demonstrated an IUD in place without an intrauterine pregnancy, as well as a clearly visualized ectopic pregnancy and free intraperitoneal fluid. Combined with a positive pregnancy test, these findings were diagnostic for ruptured ectopic pregnancy.

While there is some controversy regarding IUD use and subsequent risk for ectopic pregnancy, it is reasonable to conclude that IUD use is associated with increased risk for ectopic pregnancy, particularly in patients with a positive pregnancy test. Because the IUD was designed explicitly to prevent the implantation of intrauterine pregnancy, the diagnosis of ectopic pregnancy in this setting should be highly suspected. Our case confirms that clinicians should always consider the possibility of ectopic pregnancy in reproductive-age females even with a history of contraceptive use.

## Supplementary Information

Video 1 Transabdominal ultrasound of the pelvis. Note presence of intrauterine device within uterus and extensive pelvic hematoma (arrows).

Video 2 Transabdominal power Doppler ultrasound of the left adnexa. Note ectopic pregnancy with “ring of fire” sign reflecting peripheral hypervascularity.
